# Characterization of False or Misleading Fluoride Content on Instagram: Infodemiology Study

**DOI:** 10.2196/37519

**Published:** 2022-05-19

**Authors:** Matheus Lotto, Tamires Sá Menezes, Irfhana Zakir Hussain, Shu-Feng Tsao, Zahid Ahmad Butt, Plinio P Morita, Thiago Cruvinel

**Affiliations:** 1 Department of Pediatric Dentistry, Orthodontics and Public Health Bauru School of Dentistry University of São Paulo Bauru Brazil; 2 School of Public Health Sciences University of Waterloo Waterloo, ON Canada; 3 Department of Data Science and Business Systems School of Computing College of Engineering and Technology, SRM Institute of Science and Technology Kattankulathur India; 4 Research Institute for Aging University of Waterloo Waterloo, ON Canada; 5 Department of Systems Design Engineering University of Waterloo Waterloo, ON Canada; 6 eHealth Innovation Techna Institute University Health Network Toronto, ON Canada; 7 Institute of Health Policy, Management, and Evaluation Dalla Lana School of Public Health University of Toronto Toronto, ON Canada

**Keywords:** eHealth, fluorides, infodemiology, information seeking behavior, internet, misinformation, social media, infoveillance, health outcome, dental caries, health information, dental health

## Abstract

**Background:**

Online false or misleading oral health–related content has been propagated on social media to deceive people against fluoride’s economic and health benefits to prevent dental caries.

**Objective:**

The aim of this study was to characterize the false or misleading fluoride-related content on Instagram.

**Methods:**

A total of 3863 posts ranked by users’ total interaction and published between August 2016 and August 2021 were retrieved by CrowdTangle, of which 641 were screened to obtain 500 final posts. Subsequently, two independent investigators analyzed posts qualitatively to define their authors’ interests, profile characteristics, content type, and sentiment. Latent Dirichlet allocation analysis topic modeling was then applied to find salient terms and topics related to false or misleading content, and their similarity was calculated through an intertopic distance map. Data were evaluated by descriptive analysis, the Mann-Whitney *U* test, the Cramer V test, and multiple logistic regression models.

**Results:**

Most of the posts were categorized as misinformation and political misinformation. The overperforming score was positively associated with older messages (odds ratio [OR]=3.293, *P*<.001) and professional/political misinformation (OR=1.944, *P*=.05). In this context, time from publication, negative/neutral sentiment, author’s profile linked to business/dental office/news agency, and social and political interests were related to the increment of performance of messages. Although political misinformation with negative/neutral sentiments was typically published by regular users, misinformation was linked to positive commercial posts. Overall messages focused on improving oral health habits, side effects, dentifrice containing natural ingredients, and fluoride-free products propaganda.

**Conclusions:**

False or misleading fluoride-related content found on Instagram was predominantly produced by regular users motivated by social, psychological, and/or financial interests. However, higher engagement and spreading metrics were associated with political misinformation. Most of the posts were related to the toxicity of fluoridated water and products frequently motivated by financial interests.

## Introduction

The analysis of big data originating from people’s production and consumption of online dental information can contribute to recognizing the needs of distinct populations, aiding the planning and implementation of public health actions [1,2]. Within this context emerged the concept of infodemiology, defined as “the science of distribution and determinants of information in an electronic medium with the ultimate aim to inform public health and public policy” [3]. Specifically, internet users have adopted social media to perform queries and express their concerns, doubts, and advice about oral health conditions [4,5]. However, while these behaviors are desirable to provide empowerment and autonomy for individuals toward health education and decision-making [6,7], the content overabundance of social network ecosystems poses a challenge to the public to filter relevant posts, which leads to the consumption of false information and, consequently, the development of damaging health beliefs [8-10]. In this way, previous studies demonstrated that Instagram could be a significant source of health information, including several issues such as COVID-19 and vaccination [11], especially considering the increased popularity of this platform in recent years [12,13].

In this scenario, online false or misleading content propagates the discouragement of the consumption of fluoride-containing water and oral care products concerning their relevance, safety, and harmful consequences [14]. Notably, antifluoridation information is broadly shared on social media, deceiving people against fluoride’s economic and health benefits [15]. Moreover, some characteristics of these false or misleading posts, such as the sense of innovation and the negative sentiment charges, favor the diffusion of falsehoods in contrast to trustworthiness [16,17]. In parallel, fluoride refusal is a growing phenomenon observed in dental offices, possibly generated or reinforced by online misinformation [18]. Divergently, there is robust scientific evidence on the beneficial effects of fluoridated water, dentifrices, and mouthwashes to prevent the demineralization and promote the remineralization of dental tissues. Fluoride is considered the most effective measure to reduce the incidence and prevalence of dental caries [19-21], which is the most prevalent oral disease worldwide, affecting the permanent and deciduous teeth of approximately 2.3 billion people and 532 million children, respectively [22].

Thus, the adoption of digital strategies to manage the oral health information disorder on social media is mandatory. Toward this end, the aim of this study was to characterize the false or misleading fluoride-related content on Instagram, regarding authors’ and posts’ features, interaction and spreading metrics, and the sentiment of posts.

## Methods

### Study Design

This longitudinal and retrospective infodemiology study analyzed and characterized the false or misleading fluoride-related content of 500 English posts on Instagram. A total of 3863 posts ranked by users’ total interaction were retrieved by CrowdTangle, of which 641 were screened for the inclusion criteria. All posts were made available on Instagram between August 2016 and August 2021. Two independent investigators (ML and TSM) analyzed these posts qualitatively to define the authors’ interests, profile characteristics, content type, and sentiment of posts. Topic modeling methods were applied to find salient terms and topics related to false or misleading fluoride content. Finally, statistical analysis was performed as described in detail below.

### Ethics Considerations

This study did not require institutional review board approval from the Council of Ethics in Human Research of Bauru School of Dentistry because federal regulations do not apply to research using publicly available data that does not involve human subjects. It should be emphasized that the raw data presented in this manuscript have been anonymously disclosed in an open data repository [23].

### Search Strategy, Data Collection, and Preprocessing Data Set

CrowdTangle is an online analytics and insights tool owned by Meta Inc that enables the study of several social media metrics such as the number of posts, data, profile information, type of posts, total interaction (sum of the number of likes, comments, and views in a post), and overperforming score through specific keywords. It is also possible to access posts from distinct periods, languages, and social media, besides ranking them into various measures.

The overperforming score is a post’s performance regarding its actual interaction divided by its expected interaction according to the number of followers of the author’s profile (ie, how many ordinary followers the post reached). In this way, positive scores are associated with good performance posts, reaching a larger user’s number than simply the number of the author’s followers, and negative scores convey the opposite. Briefly, the algorithm of CrowdTangle generates benchmarks to identify these expected values using the last 100 posts from a given account. For this calculation, the top and bottom 25% posts are dropped and then the mean number of interactions are calculated with the middle 50% of posts in different time intervals (15 minutes old, 60 minutes old, 5 hours old, etc). Subsequently, when the account in question publishes a new post, the platform compares the post metrics to the calculated average and multiplies the difference by the weights in each dashboard [24].

The search strategy (“fluoride free”+”fluoride-free”) was defined from exploratory analyses of hashtags and terms related to a higher volume of posts that discouraged fluoride use on Instagram. A data set related to 3863 posts was downloaded as a CSV file on September 15, 2021, regarding specific language (English) and time frame (August 2016 to August 2021), and ranked by total interaction. The period for the collection was determined from the availability of data observed in a preliminary analysis using the search strategy on CrowdTangle and the number of worldwide Instagram users [25]. Furthermore, posts were ranked by total interaction to guarantee the inclusion of those accessed by a considerable volume of Instagram users (ie, those influencing a number of individuals not relativized by the potential of authors to achieve an audience).

Before the qualitative and natural language processing analyses, the raw data set was preprocessed in two ways depending on the type of investigation. First, the data set was screened to obtain a feasible number of posts (n=500), enabling a robust qualitative manual evaluation to feed artificial intelligence–based models, and preventing expected mischaracterization associated with automated tools. Thus, an investigator (ML) read a sample of collected posts (n=641) in full to obtain a list of the first 500 posts ranked by total interaction that satisfied the following inclusion criterion: nonrepeated false or misleading content published in English. The investigator excluded 139 posts due to repetition and 2 posts that were not published in English. It is noteworthy that this process aimed to characterize posts containing false or misleading content with the highest engagement rates on Instagram.

To ensure the quality of topic modeling analysis, another investigator (IZH) performed an additional preprocessing of the words of 500 selected posts, removing symbols, special characters, punctuations, URLs, numbers, personal pronouns, and keywords of the search strategy.

### Data Analysis

#### Qualitative Analysis

The false or misleading fluoride posts were characterized through passive qualitative analysis [26], examining information patterns and interaction metrics. This approach was directed by the most accepted definitions of the categories of information disorder, as follows: (1) misinformation, defined as false information determined based on a grounding of truth and applies only to informationally oriented content [27-29]; (2) fake news, defined as intentionally misleading and biased representational information for the benefit of the messenger sender, which contains false information, with or without a blend of one or more components of omitted important information, a decontextualized content, misleading headlines, or clickbait [30]; (3) disinformation, defined as information that is false and deliberately created to harm a person, social group, organization, or country [27,28]; and (4) conspiracy theories, which are attempts to explain the ultimate causes of significant social and political events and circumstances with claims of secret plots by two or more powerful actors [31].

Additionally, false or misleading online content can be motivated by distinct types of interest such as financial (profiting from information disorder through advertising), political (attempts to influence public opinion due to political positions), social (connecting with a particular group online or offline), and psychological (seeking prestige or reinforcement) [27]. The identification of specific motivations could be a reliable and objective indicator of authors’ intentionality, regarding that its determination is only based on the subjective judgment of online content founded on researchers’ perspectives [27]. However, according to Poe’s law, the clues left by content makers are often inadequate to differentiate between honest and dishonest mistakes (ie, the authors’ intentions to deliberately produce or share misleading content to deceive people cannot be categorically identified) [8,32]. Regarding the aforementioned difficulties to establish the specific type of information disorder, misinformation was characterized by two trained and calibrated investigators (ML and TSM) (intraclass correlation coefficient for absolute concordance varying from 0.85 to 0.92), according to the following criteria: author’s profile (regular users, business, dental office, or news agency), type of content (commercial or noncommercial), author’s interest (social, psychological, financial, and/or political), and sentiment (negative, neutral, or positive). Commercial content was detected when associated with a business, dental office, and news agency, or with regular users identified as influencers for promoting the sales of dental products. Both investigators were trained by the discussion of representative characteristics of posts. The calibration of individual judgment criteria was confirmed by the independent classification of 10% of posts (n=50). The posts that investigators divergently qualified were reassessed until consensus. Additionally, the combination of the author’s profile (dental office or others) and the detection of political interests (yes or no) defined the categories of information disorder, grouped as misinformation (posts from regular users without political interests), professional misinformation (posts from a dental office without political interests), and political misinformation (posts from authors with political interests).

#### Natural Language Processing

Topic modeling is an unsupervised machine learning method that is effectively used to identify patterns within a large corpus of unstructured documents, as previously observed in the health information area [33,34]. Interestingly, researchers who apply unsupervised algorithms do not need to previously define issues in topic modeling, corroborating with the automatized evaluation of social media data sets [35]. Besides a faster analysis, this process allows for identification that would not have been achieved by manual inspection because it is less prone to human biases [36].

We applied latent Dirichlet allocation (LDA) topic modeling using Python 3 in a Google Colab interface to determine the main salient terms and topics from the studied data set, examining the relationship between similar and different content. Synthetically, LDA is a probabilistic and word count–based model that analyzes the frequency of words to determine distinct topics [33]. Given the number of topics *K*, LDA algorithms may generate a keyword list that is most relevant to each topic individually. Although this analysis does not provide a complete meaning of social media posts, it can contribute to a good overview of issues, facilitating data interpretation [37]. A detailed description of the LDA model is provided elsewhere [38].

We defined the ideal number of topics based on the metric proposed by Nikolenko et al [39] for qualitative studies. Thus, a higher coherence score represents topic modeling with better quality, simplifying the interpretation of outputs. In this way, the coherence values were computed for *K* topics, where *K* ranges from 2 to 50, before eventually narrowing down the consideration range to 3-15 topics. We then carefully examined the models with the highest coherence values and selected that with the most significant score [35,40]. Finally, the topics’ distances were calculated to establish their similarity through an intertopic distance map.

#### Statistical Analysis

Statistical analysis was performed using the Statistical Package for Social Sciences (v. 21.0). First, the variables were dichotomized as follows: time from publication (≤859 or >859 days), categories of information disorder (misinformation or professional/political misinformation), authors’ profile (regular users or business/dental office/news agency), sentiment (negative/neutral or positive), type of content (commercial or noncommercial), type of publication (video or photo), total interaction (≤1179 or >1179), overperforming score (≤1.38 or >1.38). The continuous variables were dichotomized from their median values. Additionally, dental offices, news agency, and business profiles were dichotomized on the same side because of their common financial background.

The data normality and homogeneity were determined through the Kolmogorov-Smirnov test and Levene test, respectively. Subsequently, as data were nonnormally distributed, the comparison of total interaction and overperforming score of dichotomized variable groups was performed by the Mann-Whitney *U* test. The differences in the distribution of dichotomized variables according to the categories of information disorder were assessed by the Cramer V test.

Additionally, multiple logistic regression models were developed to evaluate the association of overperforming scores and total interaction with distinct variables. Only factors with significant Wald statistics in the simple analyses were included in the multiple regression models. For all analyses, *P*<.05 was considered significant.

## Results

As shown in Table 1, in general, the posts were predominantly commercial, produced by regular users, expressing positive sentiment, and published as an album/photo. The types of interests identified among the 500 selected posts were social (n=500, 100.0%), psychological (n=492, 98.4%), financial (n=421, 84.2%), and political (n=79, 15.8%). Considering the specific interests and authors’ profiles, the investigators categorized the posts as misinformation (n=413, 82.6%), political misinformation (n=79, 15.8%), and professional misinformation (n=8, 1.6%).

Table 1 presents the comparison of total interaction and overperforming scores with the distinct dichotomized variable groups. A significantly higher number of total interaction was found for noncommercial content items, whereas a significantly higher overperforming score was detected for >859 days, professional/political misinformation, business/dental office/news agency profiles, and negative/neutral sentiment.

**Table 1:** Comparison of total interaction and overperforming scores between dichotomized variable groups.

Variable			Posts (N=500), n (%)		Total interaction				Overperforming score					*P* values^a^			
					Mean (SD)		Median (IQR)		Mean (SD)		Median (IQR)		Total interaction			Overperforming score	
**Time from publication**														.64			<.001
	≤859 days	250 (50.0)		2720 (5031)		1149 (1265)		1.95 (7.48)		1.10 (3.22)							
	>859 days	250 (50.0)		2292 (3404)		1222 (1633)		6.15 (18.27)		1.89 (3.57)							
**Types of interest**														.36			.01
	Social, financial, and psychological	421 (84.2)		2468 (4168)		1160 (1473)		4.23 (15.20)		1.33 (4.63)							
	Social and political	79 (15.8)		2710 (4449)		1271 (1138)		3.11 (5.15)		1.98 (4.46)							
**Author’s profile**														.46			<.001
	Regular users	296 (59.2)		2701 (4759)		1189 (1486)		1.65 (13.79)		1.06 (3.05)							
	Business/dental office/news agency	204 (40.8)		2224 (3511)		1155 (1278)		7.54 (13.84)		3.59 (6.40)							
**Sentiment**														.71			.004
	Negative/neutral	77 (15.4)		2699 (5003)		1263 (1118)		3.54 (5.66)		2.30 (4.62)							
	Positive	423 (84.6)		2471 (4160)		1164 (1490)		4.15 (15.14)		1.33 (4.52)							
**Type of content**														.009			.54
	Noncommercial	95 (19.0)		3408 (5687)		1554 (2240)		1.98 (4.36)		1.63 (1.63)							
	Commercial	405 (81.0)		2295 (3878)		1144 (1279)		4.54 (15.49)		1.35 (5.02)							
**Type of publication**														.54			.61
	Video	45 (9.0)		2835 (4090)		1218 (2017)		2.26 (3.95)		1.70 (4.96)							
	Photo	455 (91.0)		2474 (4319)		1174 (1414)		4.23 (14.72)		1.38 (4.77)							

^a^Mann-Whitney *U* test (*P*<.05 considered statistically significant).

Table 2 summarizes the distribution of distinct dichotomized variable groups according to the categories of information disorder. Accordingly, the overperforming score and noncommercial content were significantly higher among professional misinformation and political misinformation groups. Furthermore, political misinformation was frequently posted by regular users with negative/neutral sentiment. By contrast, misinformation commonly presented commercial content with positive feelings.

**Table 2:** Distribution of dichotomized variable groups according to the categories of information disorder.

Variable			Misinformation (n=413), n (%)		Professional misinformation (n=8), n (%)		Political misinformation (n=79), n (%)	φ		*P* value^a^
**Time from publication**								0.79		.21
	≤859 days	209 (50.6)		6 (75.0)		35 (44.3)				
	>859 days	204 (49.4)		2 (25.0)		44 (55.7)				
**Overperforming**								0.149		.004
	≤1.38	221 (53.5)		1 (12.5)		30 (37.9)				
	>1.38	192 (46.5)		7 (87.5)		49 (62.1)				
**Author’s profile**								0.154		.003
	Regular users	247 (59.8)		0 (0)		49 (62.1)				
	Business/dental office/news agency	166 (40.2)		8 (100)		30 (37.9)				
**Sentiment**								0.712		<.001
	Negative/neutral	18 (4.3)		0 (0)		59 (74.7)				
	Positive	395 (95.7)		8 (100)		20 (25.3)				
**Total interaction**								0.061		.39
	≤1179	212 (51.3)		4 (50.0)		34 (43.1)				
	>1179	201 (48.7)		4 (50.0)		45 (56.9)				
**Type of content**								0.493		<.001
	Noncommercial	42 (10.2)		6 (75.0)		47 (59.5)				
	Commercial	371 (89.8)		2 (25.0)		32 (40.5)				
**Type of publication**								0.136		.01
	Video	31 (7.5)		0 (0)		14 (17.7)				
	Photo	382 (92.5)		8 (100)		65 (82.3)				

^a^Cramer V test (*P<*.05 considered significant).

Table 3 displays the results of the multiple logistic regression model for overperforming score. Overperforming was positively associated with older posts and professional/political misinformation. Notably, total interaction did not show significant Wald statistics for any factor in the simple analysis.

**Table 3:** Multiple logistic regression model for overperforming score (>1.38).

Variable	B^a^ (SE)	Wald statistic	OR^b^ (95% CI)	*P* value
Time from publication (>859 days)	1.192 (0.189)	39.84	3.293 (2.274-4.768)	<.001
Information disorder (professional/political misinformation)	0.664 (0.336)	3.900	1.944 (1.005-3.758)	.05
Sentiment (positive)	–0.143 (0.335)	0.163	0.867 (0.434-1.731)	.69
Constant (y-intercept)	–0.605 (0.367)	2.717	0.546	.10

^a^Unstandardized coefficient.

^b^OR: odds ratio.

**Figure 1:**
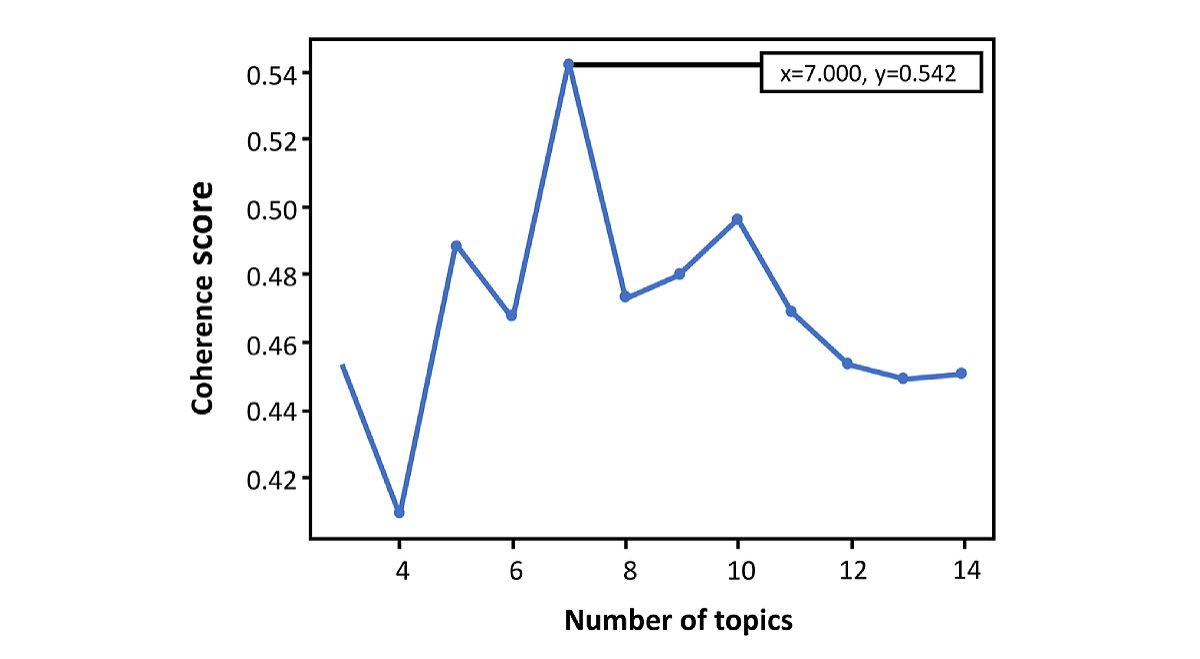
Coherence scores for distinct numbers of topics.

We adopted an exploratory process to select the topic modeling algorithm with the best performance concerning the coherence score. Figure 1 depicts these values from different number of topics, demonstrating the most significant value for 7 topics (0.54).

**Figure 2:**
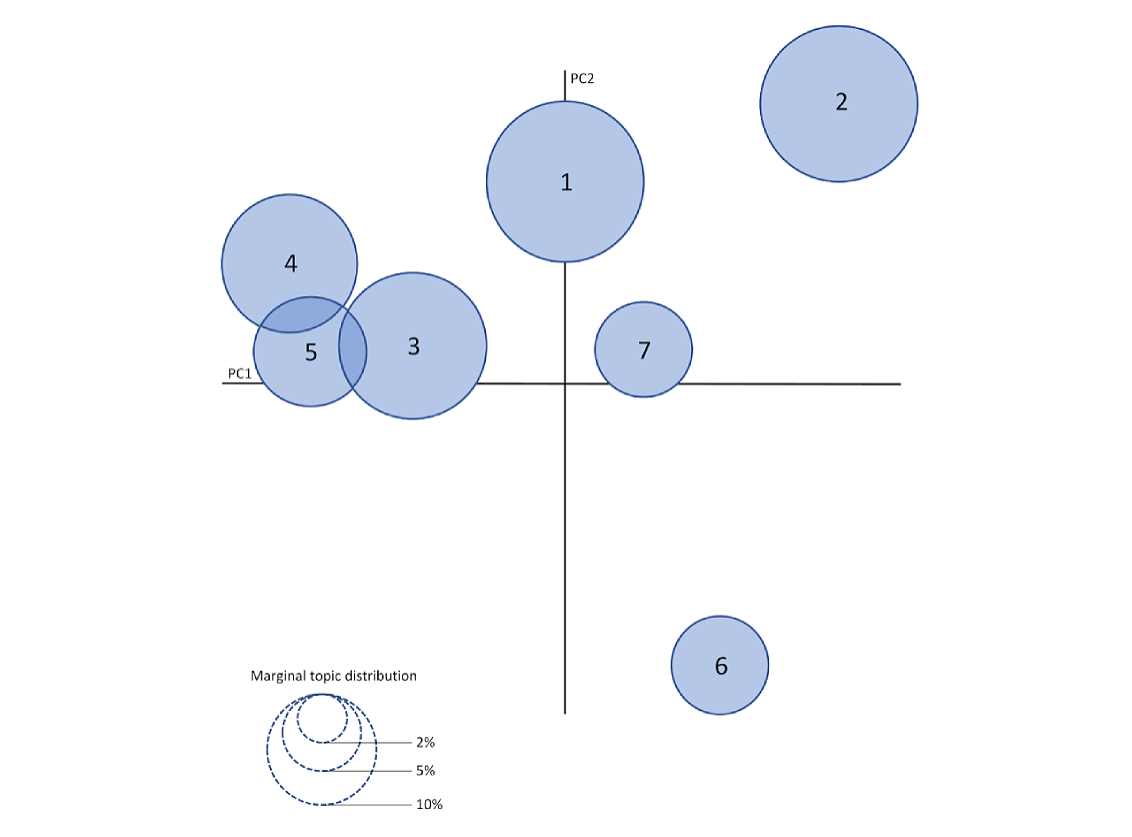
Intertopic distance map of the topic modeling analysis. Note that the bubbles are denominated according to the number of the specific topic. PC: principal component.

Thus, the LDA algorithm was executed with all posts (N=500) through the configuration *K*=7, which generated 7 different fluoride-related topics. Based on the salient keywords of each topic, we attributed a brief description to determine their meaning and subsequently stratified them regarding the main issues, as presented in Table 4. Figure 2 shows the topics’ distances to establish their similarity through an intertopic distance map. There was higher proximity of topics 3, 4, and 5; a similarity between topics 1 and 7; and a considerable distance of topics 2 and 6 from the others. Overall, the topics that emerged from the analysis were related to discouraging the consumption of fluoridated products and water by adults and children, justified by their toxicity, using arguments on the improvements of oral health habits (topics 1 and 7), side effects of fluoride (topic 2), the use of dentifrice containing natural and/or vegan ingredients (topics 3, 4, and 5), and propaganda of fluoride-free oral care products (topic 6).

The most representative words of each topic were employed to determine its issues, depending on the specific context of posts, as verified in the manual analysis. For example, the word “giveaway” was linked to posts about dentifrices containing natural and/or vegan ingredients because several authors promote draws of this kind of products, as follows: “It is GIVEAWAY TIME! Baby care is simplified with Dr. Brown’s wide range of health and hygiene products.”

**Table 4:** Fluoride-related salient topics stratified according to number of posts, most frequent words, issues, and examples.

Topic	Posts, n	Most frequent words	Issues	Examples
1	115	Love, Day, Use, Product, Body, Skin, Time, Natural, Get, Feel, Help, Work, Life, Know, Try	Improvements of oral health habits	“Do your kids enjoy brushing their teeth?! My boys used to fight it until we made it a fun routine!! @grinnatural has now become part of our routine and not only has it helped our kids oral care but has also become a fun activity they look forward to!”
2	62	Water, Drink, Health, Body, Level, Use, Filter, Pineal Gland, Study, High, Know, Brain, Cause, Bone, Source	Side effects of fluoride	“Intentional poisoning of the municipal water sources with toxic fluoride and other toxins/heavy metals of primary source for pineal gland calcification”
3	32	New, Ingredient, Love, Ad, Clean, Formula, Kid, Fresh, Target, Adult, Know, Try, Smile, Toothpaste, Flavor	Use of dentifrice containing natural/vegan ingredients	“Brush-brush the germs away from your baby’s teeth with the help of Mee Mee’s Fluoride Free Strawberry Flavour Toothpaste”
4	93	Natural, Whiten, Charcoal, Product, Smile, Activate Charcoal, Use, Vegan, Giveaway, Ingredient, White, Toothbrush, Winner, Follow, Coconut	Use of dentifrice containing natural/vegan ingredients	“The Grounded Activated Charcoal Teeth Powder is a 100% natural and fluoride free teeth whitening formula to brighten your teeth shade, remove plaque, cleanse the mouth, remove toxins & make your mouth feel sparkling clean”
5	77	Kid, Brush, Brush Tooth, Love, Baby, Fun, Toothbrush, Fruit, Natural, Ad, Flavor, Routine, Start, Child, Safe Swallow	Use of dentifrice containing natural/vegan ingredients	“#ad Chloe’s favorite part of her morning routine is brushing her teeth. Thankfully @toms_of_maine makes brushing her teeth fun with their Silly Strawberry toothpaste. Chloe loves the delicious taste and I love that it’s natural free from artificial flavors, colors and preservatives”
6	93	Oral Care, Gum, Mouth, Product, Mouthwash, Bacteria, Disease, Cavity, Oral, Plaque, Bad, Natural, @Garnensgarden, Breath, Garnens Garden	Propaganda of fluoride-free oral care products	“Make the Switch, to an all-natural oral care products from Garners Garden (@garnersgarden)! Protect your gums and teeth from cavities and bad bacteria!”
7	28	Organic, Use, Tongue, Add, Oil, Healthy, Daily, Toxin, Routine, Clean, Coconut Oil, Brush, Day, Tap, and Antibacterial	Improvements of oral health habits	“How many of you guys Oil Pull? It's one of my favorite ways to detox and keep my teeth healthy/white”

## Discussion

### Principal Findings and Comparison With Prior Work

These findings indicate that the predominant false or misleading fluoride Instagram posts were categorized as misinformation (n=413) and political misinformation (n=79). In this context, several characteristics were related to the increment of overperforming scores of messages, such as time from publication, negative or neutral sentiment, business/dental office/news agency author’s profile, and social and political interests. In particular, older messages (odds ratio [OR]=3.29) and professional/political misinformation (OR=1.94) were associated with better performance of spreading among Instagram users. Remarkably, commercial content was significantly more prevalent in the misinformation category than in the professional and political misinformation categories. Furthermore, regular users preponderantly published political misinformation presenting negative or neutral sentiment, whereas misinformation was linked to positive commercial posts. The messages generally addressed the toxicity of fluoridated products and water, focusing on improving oral health habits, side effects of fluoride, dentifrice containing natural and/or vegan compounds, and propaganda of fluoride-free oral care products. Although previous studies have analyzed fluoride-related information on social media, including Instagram [13-15,17,41], this study differs regarding only focusing on analyses of false or misleading fluoride information, identified based on contemporary concepts and methods on information disorder.

From these outcomes, we confirmed that oral health information seekers engage more with political fluoride misinformation, even after excluding the influence of time as a confounding factor. Indeed, social media consumers tend to connect with others similar to themselves regarding political ideology [42]. People motivated by specific political overviews, influenced by personal characteristics such as beliefs and values, are predisposed to be more interactive with congruent arguments and assimilate them uncritically (confirmation bias) [43,44]. Thus, greater political homophily is associated with increased user interaction since it reinforces similar ideologies [45]. It is important to note that individuals are susceptible to believing and sharing misinformation regardless of their underlying political creed [44].

Moreover, LDA topic modeling categorized most of the political misinformation in topic 2, covering the possible side effects from fluoride toxicity, as exemplified by the following posts:

over three hundred studies have found that fluoride is literally a neurotoxin

fluoridated water provides no benefits, only risks. Babies given fluoridated water in their formula may have reduced IQ scores.

This demonstrates that Instagram users were strongly influenced by concerns and fears surrounding fluoridated products and the water supply, interacting with negative sentiment posts that emphasized the adverse health aspects of fluoride. These outcomes are in agreement with posts of Twitter users [17].

The positive impact of the time of availability of posts on overperforming scores is an expected result because users have more opportunities to access these posts in comparison to more recent posts. Likewise, authors’ profiles linked to economic activities, such as companies, dental offices, and news media, usually structure their messages to attract customers, besides probably paying money to promote their content on Instagram, which increases people’s engagement and thus raises content diffusion. Surprisingly, we detected financial interest in most posts, including a substantial portion of regular users (digital influencers) that publicized fluoride-free products. Moreover, several salient topics that emerged from modeling were closely connected to brands. Indeed, the distribution of information disorder often has a close relationship with economic gains [27]. Specifically, our findings suggest that the antifluoridation proposals strongly connect with financial concerns beyond the above-discussed ideological aspects. In this sense, distinct oral care companies have been focused on developing products that meet the individual wishes of consumers, even with the absence of scientific evidence [46].

### Practical Implications

These findings can support the development of methods and models to automatically identify false or misleading content items and assess their propagation on social media. In addition, outcomes such as topic modeling can subside the elaboration of eHealth and mobile health fluoride-related educational approaches to guide social media users toward the consumption of adequate online oral health information [47]. In this context, dental professional teams need to be conscious of fluoride-related misinformation toward improving the quality of their relationship with patients. Additionally, universal access to oral health, improving eHealth and electronic literacy, and offering high-quality dental information are desirable to prevent the consumption of deceptive messages. Certainly, policymakers should recognize the negative influence of these false posts on communities, creating guidelines and laws to control the spread of information disorder. Specifically, social media managers should be encouraged to develop mechanisms for screening posts to detect false or misleading content before considering messages eligible for sponsorship, avoiding the dissemination of misinformation. Despite the difficulties in determining the authors’ intentions, society needs to start discussing education measures and possible penalties for misinformation propagators, within the confines of democratic values, mainly when disseminated by health professionals.

### Limitations

This study has some limitations. First, we collected the sample from a specific search strategy composed of two keywords, limiting the findings’ generalization to all false or misleading fluoride content. However, we performed an exploratory analysis to determine the most representative keywords with the greatest spread for the thematic analysis in data collection. Second, the two independent investigators analyzed only 500 posts due to work restrictions associated with human analysis, in accordance with previous dental studies [4]. In addition, the manual labeling of data sets is imperative to training artificial intelligence models for natural processing language tasks, ensuring high accuracy and data generalizability [48]. Third, as previously described, we cannot differentiate misinformation from other types of information disorder because of the incapacity of determining authors’ intentionality objectively and precisely [49]. Notwithstanding, the characterization of misinformation was improved, verifying the association of specific interests and authorship with interaction metrics. Fourth, these interpretations were based on content published in English. Although English is the most spoken language worldwide, cultural aspects likely influenced the detection of falsehoods.

### Conclusions

False or misleading fluoride posts available on Instagram were predominantly characterized as misinformation produced by regular users motivated by social, psychological, and/or financial interests; however, misinformation with social and political interests was associated with higher engagement and spreading metrics. In general, the content of posts was related to the toxicity of fluoridated water and products, frequently motivated by financial interests.

## Abbreviations

LDA: latent Dirichlet allocation
OR: odds ratio
